# Genetic variations in *ACE2* gene associated with metabolic syndrome in southern China: a case–control study

**DOI:** 10.1038/s41598-024-61254-5

**Published:** 2024-05-07

**Authors:** Min Pan, Mingzhong Yu, Suli Zheng, Li Luo, Jie Zhang, Jianmin Wu

**Affiliations:** 1 Department of Geriatrics, The First Affiliated Hospital of Fujian Medical University, Fuzhou, 350005 Fujian People’s Republic of China; 2 Department of Geriatrics, National Regional Medical Center, Binhai Campus of the First Affiliated Hospital, Fujian Medical University, Fuzhou, 350005 Fujian People’s Republic of China; 3 Branch of National Clinical Research Center for Aging and Medicine, The First Affiliated Hospital of Fujian Medical University, Fuzhou, 350005 Fujian People’s Republic of China; 4 Clinical Research Center for Geriatric Hypertension Disease of Fujian Province, The First Affiliated Hospital of Fujian Medical University, Fuzhou, 350005 Fujian People’s Republic of China; 5 Fujian Hypertension Research Institute, Fuzhou, 350005 Fujian People’s Republic of China

**Keywords:** Metabolic syndrome, Angiotensin-converting enzyme 2, Gene polymorphism, Obesity, Gender heterogeneity, Genotype, Metabolic syndrome

## Abstract

Metabolic syndrome (MetS) is closely related to cardiovascular and cerebrovascular diseases, and genetic predisposition is one of the main triggers for its development. To identify the susceptibility genes for MetS, we investigated the relationship between angiotensin-converting enzyme 2 (ACE2) single nucleotide polymorphisms (SNPs) and MetS in southern China. In total, 339 MetS patients and 398 non-MetS hospitalized patients were recruited. Four *ACE2* polymorphisms (rs2074192, rs2106809, rs879922, and rs4646155) were genotyped using the polymerase chain reaction-ligase detection method and tested for their potential association with MetS and its related components. *ACE2* rs2074192 and rs2106809 minor alleles conferred 2.485-fold and 3.313-fold greater risks of MetS in women. *ACE2* rs2074192 and rs2106809 variants were risk factors for obesity, diabetes, and low–high-density lipoprotein cholesterolemia. However, in men, the *ACE2* rs2074192 minor allele was associated with an approximately 0.525-fold reduction in MetS prevalence. Further comparing the components of MetS, *ACE2* rs2074192 and rs2106809 variants reduced the risk of obesity and high triglyceride levels. In conclusion, *ACE2* rs2074192 and rs2106809 SNPs were independently associated with MetS in a southern Chinese population and showed gender heterogeneity, which can be partially explained by obesity. Thus, these SNPs may be utilized as predictive biomarkers and molecular targets for MetS. A limitation of this study is that environmental and lifestyle differences, as well as genetic heterogeneity among different populations, were not considered in the analysis.

## Introduction

Metabolic syndrome (MetS) is a general term for a series of complex metabolic disorders, including obesity, insulin resistance, hypertension, dyslipidemia, endothelial dysfunction, and chronic stress^1^. MetS seriously endangers human health, as the risks of myocardial infarction and stroke in MetS patients were 1.80 and 2.05 times greater than those in people without MetS, respectively^2^. Genetic factors are a significant part of the cause of MetS, and the heritability of MetS is between 10 and 30%^3,4^. Although various treatments for MetS have been proposed, their efficacy remains limited, with one possible reason being genetic heterogeneity between patients^5^.

Genetic factors contribute to the development of metabolic diseases such as obesity, hypertension, type 2 diabetes, and dyslipidemia; therefore, genetic testing can guide clinical medication^6–8^. However, only a limited number of studies have explored the genetic influence on MetS development. Loredan et al. showed that *APOA5* gene polymorphism is an independent risk factor for the development of MetS^9^; however, this observation was based on a small sample size. In a meta-analysis of genetic polymorphisms, there was a high level of unexplained heterogeneity between the *APOC3* C482T polymorphism and MetS^10^. At the same time, the role of genetic variants in some clinical variables that have been shown to be closely related to MetS remains unknown.

Angiotensin-converting enzyme 2 (ACE2) antagonizes the classic angiotensin-converting enzyme-angiotensin II-angiotensin type 1 receptor axis, with vasodilatory, anti-fibrotic, cardioprotective, and anti-inflammatory^11^. Deficiency or suppression of *ACE2* may lead to hypertension, whereas its overexpression or activation can prevent it^12^. Bindom et al. confirmed that *ACE2*-targeting therapy can improve insulin resistance in diabetic mice by suppressing pancreatic β cell apoptosis, thus reducing blood glucose levels^13^. Besides, *ACE2* knockout mice have shown progressive reduced insulin levels and impaired glucose tolerance^14^. The association of *ACE2* gene variants with hypertension as well as diabetes has recently been reported. Five single nucleotide polymorphisms (SNPs) of *ACE2* were shown to affect blood pressure levels in women, implicating a role for *ACE2* in the genetic basis of essential hypertension^15^. In addition, one study showed that *ACE2* SNPs were associated with diabetes and diabetes-related cardiovascular complications^16^. Patel et al. also demonstrated that *ACE2* gene variants can increase the risk of hypertension in Caucasian patients with type 2 diabetes^17^.

As diabetes mellitus and hypertension are major conditions associated with MetS, we inferred that polymorphisms in *ACE2* might affect MetS incidence. Therefore, we aimed to explore the relationship between *ACE2* polymorphisms and MetS through genotyping and analysis of the *ACE2* locus. Our findings are expected to inform a new strategy for screening high-risk groups for MetS and precision medicine approaches.

## Results

### Comparison of clinical features between MetS group and non-MetS group

MetS is a cluster of conditions that increase your risk of cardiovascular disease and stroke, which is influenced by a variety of factors. For this, we explored a variety of clinical data such as baseline characteristics, medical history, and MetS composition for 339 MetS patients and 398 participants in the non-MetS group, including 453 men and 284 women, as shown in Table 1. Age, BMI, WC, SBP, heart rate, and serum concentrations of triglycerides, VLDL-C, FPG, 2-h postprandial plasma glucose, and HbA1c were higher in MetS patients (*P* < 0.05). Further, MetS cases exhibited lower levels of HDL-C, LDL-C, ApoA1 and ApoA1/ApoB (*P* < 0.05). No differences were observed concerning sex, duration of hypertension, smoking status, drinking status, DBP, and total cholesterol level (*P* > 0.05, see Table 1).

**Table 1:** Comparison of clinical characteristics of MetS and non-MetS patients.

Variables	Overall (N = 737)	Non-MetS (N = 398)	MetS (N = 339)	*P*
General characteristics				
Age (year)	60.97 ± 12.92	59.21 ± 13.89	63.04 ± 11.35	< 0.001
Sex (%)				0.392
Men	453 (61.47)	239 (52.75)	214 (47.25)	
Women	284(38.53)	159 (55.98)	125 (44.02)	
Hypertension (%)	476 (64.59)	169 (42.46)	307 (90.56)	< 0.001
Hypertension duration (year)	8.00 (3.00, 10.00)	7.00 (2.00, 10.00)	8.00 (3.00, 10.00)	0.268
Diabetes (%)	246 (33.38)	42 (10.55)	204 (60.18)	< 0.001
Diabetes duration (year)	3.00 (0.00, 7.00)	0.50 (0.00, 6.00)	3.00 (0.75, 8.00)	0.018
Antihypertensive drugs (%)	365 (49.53)	128 (32.16)	237 (69.91)	< 0.001
Hypoglycemic drugs (%)	141 (19.13)	15 (3.77)	126 (37.17)	< 0.001
Smoking status (%)	177 (24.02)	98 (24.62)	79 (23.30)	0.676
Drinking status (%)	96 (13.03)	47 (11.81)	49 (14.45)	0.288
Metabolism project				
TC (mmol/l)	4.25 ± 0.98	4.29 ± 0.94	4.21 ± 1.02	0.246
TG (mmol/l)	1.53 ± 1.08	1.15 ± 0.59	1.98 ± 1.32	< 0.001
HDL (mmol/l)	1.07 ± 0.28	1.19 ± 0.29	0.94 ± 0.22	< 0.001
LDL (mmol/l)	2.73 ± 0.88	2.80 ± 0.84	2.64 ± 0.93	0.011
VLDL (mmol/L)	0.70 ± 0.49	0.52 ± 0.27	0.90 ± 0.60	< 0.001
ApoA1 (mmol/L)	1.31 ± 0.22	1.36 ± 0.22	1.25 ± 0.20	< 0.001
ApoB (mmol/L)	0.92 ± 0.23	0.91 ± 0.22	0.93 ± 0.23	0.180
ApoA1/ApoB (%)	1.50 ± 0.42	1.57 ± 0.42	1.42 ± 0.41	< 0.001
HbA1c (%)	6.24 ± 1.16	5.77 ± 0.78	6.61 ± 1.27	< 0.001
FPG (mmol/L)	5.22 ± 1.45	4.77 ± 0.86	5.74 ± 1.79	< 0.001
PPG (mmol/L)	8.46 ± 5.31	7.18 ± 3.27	9.67 ± 6.47	< 0.001
Anthropometric signs				
Height (cm)	164.43 ± 7.75	163.77 ± 7.75	165.19 ± 7.70	0.013
Weight (kg)	65.77 ± 11.44	61.22 ± 9.51	71.12 ± 11.21	< 0.001
BMI (kg/m^2^)	24.27 ± 3.49	22.80 ± 2.99	25.99 ± 3.24	< 0.001
WC (cm)	87.03 ± 9.86	82.04 ± 8.01	92.88 ± 8.54	< 0.001
SBP (mm Hg)	128.87 ± 14.57	127.45 ± 13.62	130.55 ± 15.47	0.004
DBP (mm Hg)	73.03 ± 9.72	72.99 ± 9.35	73.09 ± 10.15	0.894
HR (beats/min)	69.25 ± 14.20	67.83 ± 14.60	70.98 ± 13.52	0.004

MetS, metabolic syndrome; SNPs, single nucleotide polymorphisms; TC, total cholesterol; TG, total triglyceride; HDL, high-density lipoprotein cholesterol; LDL, low-density lipoprotein cholesterol; VLDL, very low-density lipoprotein cholesterol; HbA1c, glycosylated hemoglobin; FPG, fasting plasma glucose; PPG, postprandial plasma glucose; BMI, body mass index; WC, waist circumference; SBP, systolic blood pressure; DBP, diastolic blood pressure; HR, heart rate.

### Genotype and allelic frequencies of ACE2 tagSNPs

In women, the frequency of the *ACE2* rs2074192 and rs2106809 minor alleles in MetS cases was significantly higher than in the non-MetS group (*P* < 0.05, Table 2). However, we did not observe any differences in the frequencies of rs879922 or rs4646155 between MetS and non-MetS groups (Table 2).

**Table 2:** Genotype and allele frequencies of *ACE2* tagSNPs in MetS patients.

Group	Genotype			*P*^a^	Allele		*P*
rs2074192	CC	CT	TT		C	T	
Women							
MetS (n = 125)	35 (24.0)	67 (58.9)	23 (17.1)	0.687	137 (54.8)	113 (45.2)	0.008
Non-MetS (n = 159)	72 (45.3)	65 (40.9)	22 (13.8)		209 (65.7)	109 (34.3)	
Men							
MetS (n = 214)	–	–	–	–	166 (77.6)	48 (22.4)	0.005
Non-MetS (n = 239)	–	–	–	–	157 (65.7)	82 (34.3)	
rs2106809	GG	GA	AA		G	A	
Women							
MetS (n = 125)	27 (21.6)	80 (64.0)	18 (14.4)	0.052	134 (53.6)	116 (46.4)	< 0.001
Non-MetS (n = 159)	75 (47.2)	69 (43.4)	15 (9.4)		219 (68.9)	99 (31.1)	
Men							
MetS (n = 214)	–	–	–	–	160 (74.8)	54 (25.2)	0.176
Non-MetS (n = 239)	–	–	–	–	165 (69.0)	74 (31.0)	
rs879922	GG	GC	CC		G	C	
Women							
MetS (n = 125)	0 (0.0)	6 (4.8)	119 (95.2)	0.693	6 (2.4)	244 (97.6)	0.875
Non-MetS (n = 159)	0 (0.0)	7 (4.4)	152 (95.6)		7 (2.2)	311 (97.8)	
Men							
MetS (n = 214)	–	–	–	–	2 (0.9)	212 (99.1)	0.204
Non-MetS (n = 239)	–	–	–	–	6 (2.5)	233 (97.5)	
rs4646155	CC	CT	TT		C	T	
Women							
MetS (n = 125)	120 (96.0)	5 (4.0)	0 (0.0)	0.739	245 (98.0)	5 (2.0)	0.923
Non-MetS (n = 159)	153 (96.2)	6 (3.8)	0 (0.0)		312 (98.1)	6 (1.9)	
Men							
MetS (n = 214)	–	–	–	–	211 (98.6)	3 (1.4)	0.815
Non-MetS (n = 239)	–	–	–	–	235 (98.3)	4 (1.7)	

^a^*P* value of Hardy–Weinberg equilibrium detection.

In men, compared with non-mets participants, the frequency of the *ACE2* rs2074192 minor allele was significantly lower in MetS cases (*P* < 0.05; Table 2). No differences in the association of *ACE2* tagSNPs with MetS risk were found for rs2106809, rs879922, and rs4646155 (Table 2). In addition, the results of linkage disequilibrium of *ACE2* tagSNPs were shown in supplementary materials, Figure S1 and Table S1.

### Association of ACE2 tagSNPs with MetS

**Figure 1:**
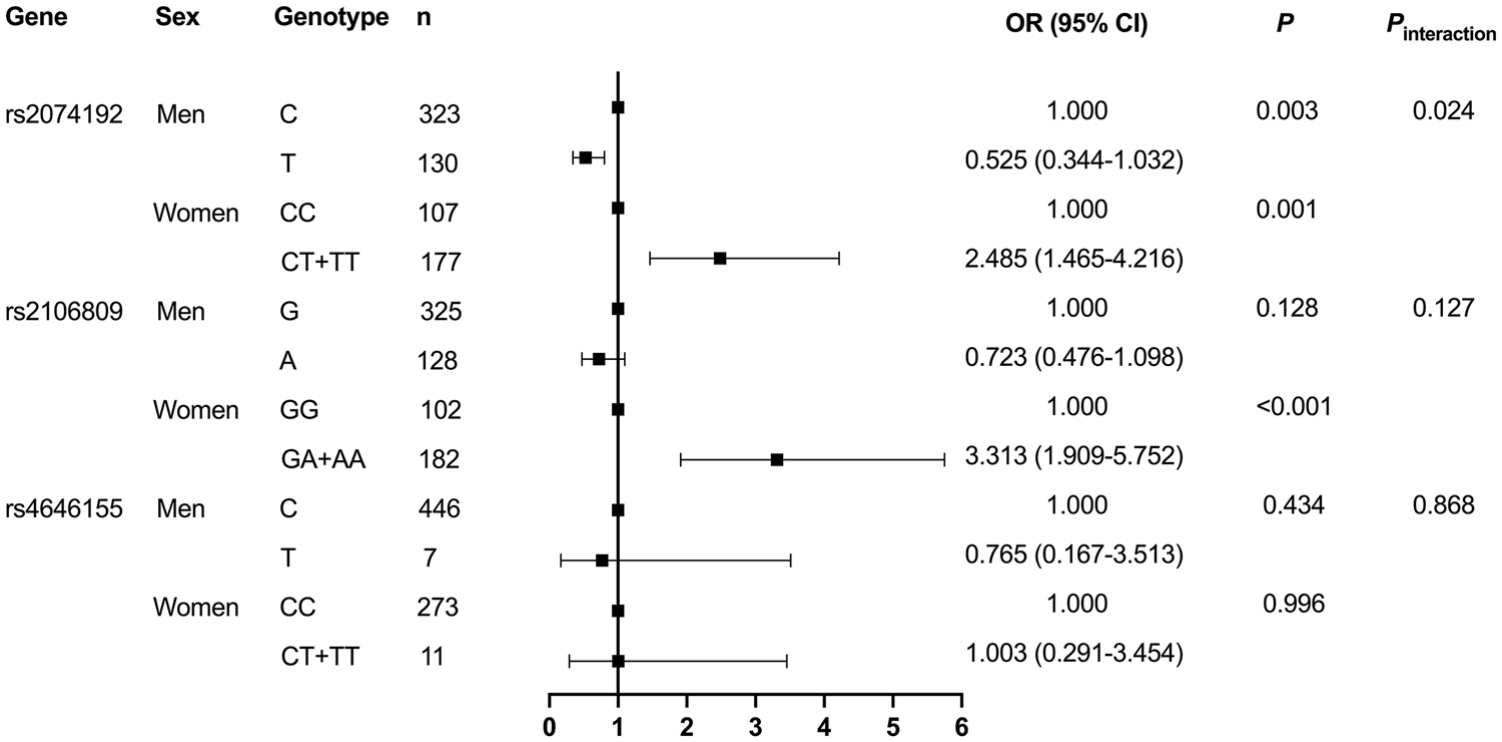
Comparison of OR values of MetS caused by different genotypes. Binary logistic regression analysis showed that *ACE2* rs2074192 was a protective factor against MetS in men, whereas *ACE2* rs2074192 and rs2106809 were risk factors for MetS in women, after adjusting for factors such as age, smoking, and alcohol consumption. There was significant sexual heterogeneity in the risk of MetS with minor alleles of *ACE2* rs2074192. OR, odds ratio; CI, confidence interval.

A multivariate logistic regression analysis was used to assess the association between genetic polymorphisms and the risk of MetS. Our results showed that, after adjusting for age, smoking and drinking status, the minor alleles of *ACE2* rs2074192 and rs2106809 increased the risk of MetS in women by 2.485 and 3.313 times, respectively (both *P* < 0.05, Fig. 1). Neither rs879922 nor rs4646155 was associated with MetS risk.

After adjusting for age, smoking and drinking status in men, the *ACE2* rs2074192 minor allele was associated with lower MetS risk (*P* < 0.05; Fig. 1). However, no association was observed for rs2106809, rs879922, and rs4646155. the The risk of MetS in participants with *ACE2* rs2074192 alleles showed significant sex-heterogeneity by interaction analysis (*P*_interaction_ < 0.05; Fig. 1).

### Association between ACE2 gene polymorphism and the number of MetS components

**Figure 2:**
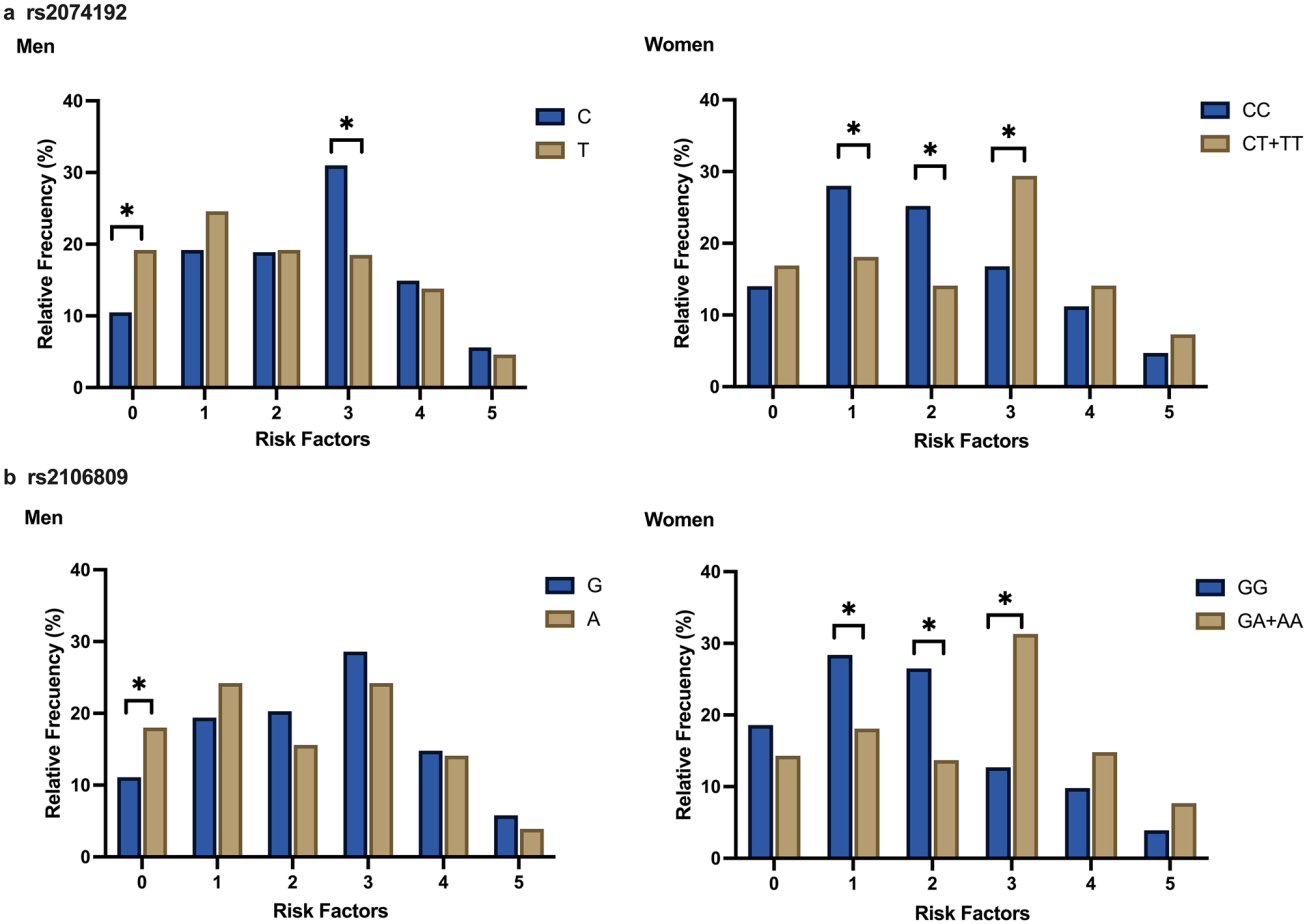
Comparison of the number of MetS components caused by different genotypes of rs2074192 (**a**) and rs216809 (**b**). Men with the rs2074192 (T) and rs2106809 (A) mutations were more likely to have no MetS components. In women, rs2074192 (CT + TT) and rs2106809 (GA + AA) were associated with an increase in the number of MetS components. * *P* < 0.05.

As shown in Fig. 2, an increased number of MetS components were observed in women with *ACE2* rs2074192 (CT + TT, *P* < 0.05) and rs2106809 (GA + AA, *P* < 0.05). Male patients with the *ACE2* rs2074192 (T, *P* = 0.013) and rs2106809 (A, *P* = 0.049) alleles were more likely to exhibit no MetS components.

### Risk comparison of ACE2 rs2074192 and rs2106809 genotypes concerning different components of MetS

**Figure 3:**
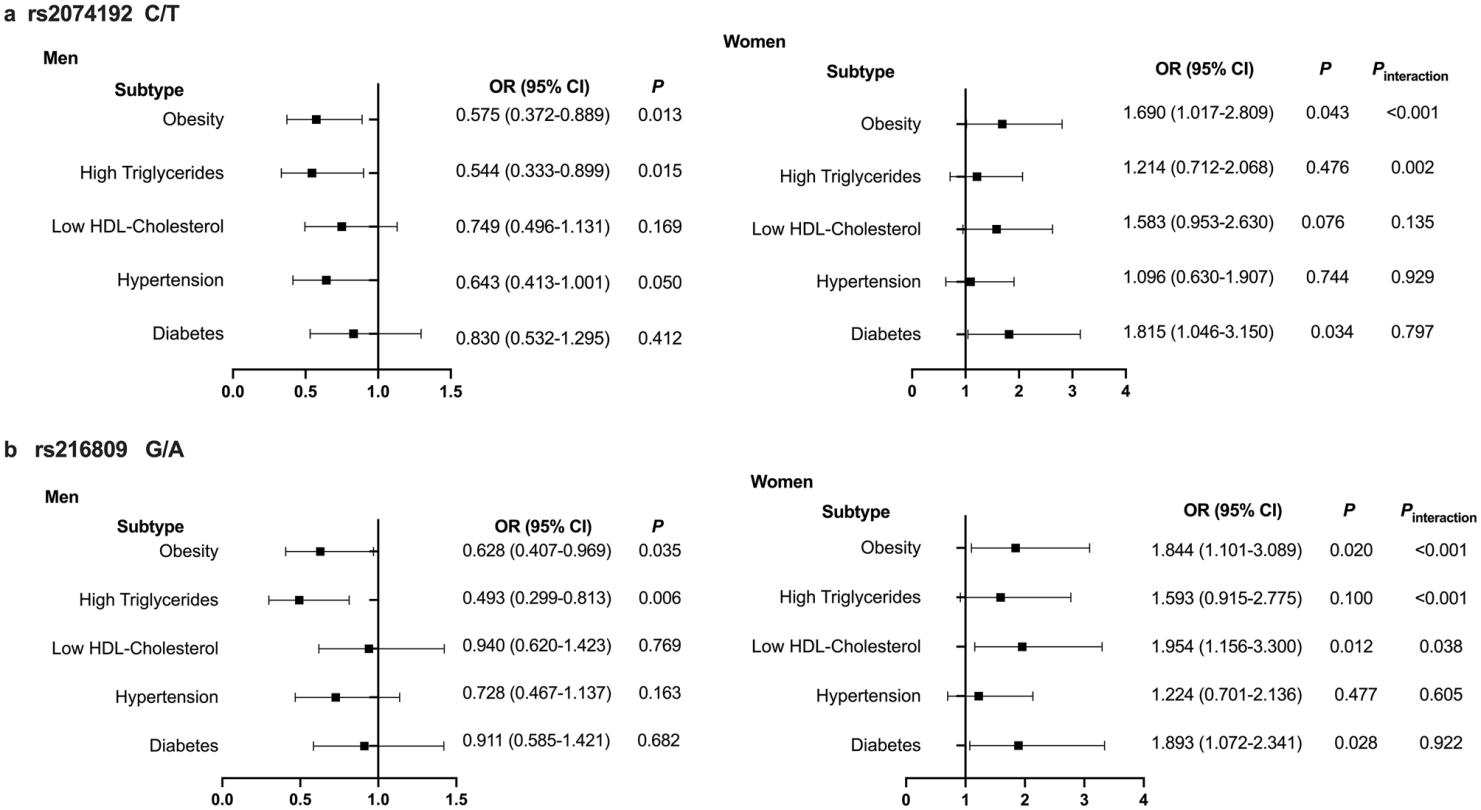
Comparison of the incidence of MetS components in different allelic genotypes of rs2074192 (**a**) and rs216809 (**b**). The rs2074192 (CT + TT) and rs2106809 (GA + AA) alleles were associated with a higher risk of obesity, diabetes, and low levels of high-density lipoprotein cholesterol in women, while the rs2074192 (T) and rs2106809 (A) alleles were associated with lower risk of obesity and hypertriglyceridemia in men, after adjusting for age, smoking, and alcohol consumption. There was significant heterogeneity identified in risk for obesity and hypertriglyceridemia by sex.

In women, the *ACE2* rs2074192 (CT + TT) and rs2106809 (GA + AA) alleles were associated with a higher risk of developing obesity, diabetes, and low levels of HDL-C. Male patients with the *ACE2* rs2074192 (T) and rs2106809 (A) variants had a lower risk of developing obesity and hypertriglyceridemia (all *P* < 0.05, after adjusting for age, smoking and drinking status, respectively, Fig. 3).

Furthermore, the prevalence of obesity and hypertriglyceridemia in participants with rs2074192 and rs216809 minor alleles was significantly different between men and women, while the risk of low levels of HDL-C was significant heterogeneity identified in patients with rs216809 variants (P_interaction_ < 0.05, respectively).

## Discussion

In this study, we investigated the genetic polymorphisms of four *ACE2* loci in southern China to determine the effect of *ACE2* variation on MetS susceptibility. Our results showed that *ACE2* SNPs rs2074192 and rs2106809 were closely related to MetS and its components in the southern Chinese population. The effect of genetic diversity was sex-specific, as women harboring the *ACE2* rs2074192 and rs2106809 SNPs may be at a higher risk of developing MetS than men. In addition, *ACE2* rs2074192 and rs2106809 gene variants were associated with obesity in both men and women.

In recent years, many studies have explored the key genetic loci of metabolic syndrome. A study from Pakistan showed that SNP rs1333049 at the 9p21 locus significantly increased MetS risk and could be used as a genetic predictor of MetS^18^. Yeh et al. explored the genetic correlation of *APOE* loci with MetS in Taiwan Biobank participants. Genotype–phenotype association analysis showed that *APOE* rs429358 and *APOC1* rs438811 were significantly associated with MetS, highlighting the key role of *APOE* and *APOC1* variants in predicting MetS^19^. A genome-wide association study from South Korea showed that rs662799, located in *APOA5*, significantly correlated with MetS after adjusting for age and sex^20^. In our study, the *ACE2* rs2074192 T and rs2106809 A alleles were associated with MetS risk in women, increasing it by 2.485-fold and 3.313-fold, respectively.

This association of *ACE2* gene variants on MetS risk was sex-specific. In men, we found that the *ACE2* rs2074192 T allele was indicative of lower MetS risk. Such sex-specific differences are common when it comes to the genetic basis of certain conditions. A study on the relationship between *ACE2* variants and left ventricular hypertrophy revealed that the minor alleles of *ACE2*, rs2074192, and rs2106809, increased susceptibility to left ventricular hypertrophy in women, but not in men^21^. Another study explored the role of *ANK1* SNP rs516946 in the relationship between dietary iron level and MetS, finding an interaction with the association between MetS and dietary iron in Chinese males, but not in females^22^. One possible reason for the sex-specific difference in our results is that *ACE2* is located on the X chromosome, so the number of alleles varies significantly between sexes. Another possible explanation is that sex hormones differentially affect tissue gene expression, leading to sex-specific disease susceptibility^23^. An increasing body of evidence suggests that *ACE2* expression is regulated by sex hormones, which gives rise to sex-specific differences^24,25^. Furthermore, Silander et al. showed that loci associated with cardiovascular disease risk are more frequently detected in women, whereas men are more susceptible to environmental and lifestyle risk factors^23^. Owing to the difference in sex-specific heritability, the potential effect of gene variants on clinical diseases may be gender-specific. There are gender differences in coronary heart disease- and metabolic syndrome-associated SNP heritability, suggesting that gender-gene interaction patterns may shed light on underlying genetic susceptibility^26^. A large-scale genetic association study of metabolic syndrome in patients with coronary heart disease revealed that several gene variants exhibited significant gender-gene interactions, demonstrating that the genetic effect was stronger in females^27^. In addition, differences in MetS prevalence have been noted in NHANES surveys in the United States. The prevalence of MetS is significantly higher in African American women, suggesting that gender-related heritability also varies among different ethnic groups^28^. Another survey also showed that gender differences in the prevalence of MetS vary by ethnicity: among the white ethnic group, the prevalence is nearly twice as high in men as in women, whereas among black and Mexican American ethnic groups, MetS is more prevalent in women^29,30^.

In our study, BMI, WC, systolic blood pressure, serum triglycerides, and blood glucose levels were significantly elevated in the MetS group, which is representative of MetS. Each individual factor negatively impacts human health. It is important to note, however, that MetS is more than a simple combination of these factors. MetS is associated with endothelial dysfunction, a chronic stress state, and other metabolic abnormalities; therefore, the negative impact of MetS on human health is greater than that of the sum of all factors. In addition, women with the *ACE2* rs2074192 T and rs2106809 A genotypes tended to have more risk factors for MetS, with further analysis revealing a higher risk of diabetes, obesity, and low HDL cholesterol levels. In addition, men with *ACE2* rs2074192 T and rs2106809 A alleles tended to have lower rates of obesity and elevated triglyceride levels. Our results are partially consistent with those of a previous study on a diabetic Uygur population that reported a close correlation between *ACE2* rs2074192 and type 2 diabetes mellitus^16^. Another study on risk genes for gestational diabetes revealed that the *ACE2* rs2074192 polymorphism increased the risk of developing gestational diabetes^31^. A study on the interaction of genes from the renin-angiotensin system with type 2 diabetes showed that a combination of multisite genetic variants, including *ACE2* rs2106809, was associated with a higher risk, further supporting the link between *ACE2* and diabetes^32^. In addition, a study from Spain showed that *ACE2* polymorphisms were associated with obesity and hyperlipidemia in female adolescents, suggesting that the *ACE2* SNP rs2074192 may confer susceptibility to obesity and hyperlipidemia in women^33^. Another study found that *ACE2* rs2106809 variants may lead to lower HDL-C levels^34^. Obesity is an important component of MetS, with the abnormal fat metabolism and insulin resistance caused by obesity being critical factors in MetS development^35^. As these *ACE2* variants are associated with obesity in both sexes, it is speculated that *ACE2* rs2074192 and rs2106809 polymorphisms may influence the potential risk of obesity-associated MetS.

At the same time, our current data did not reveal an association of *ACE2* rs2074192 and rs2106809 SNPs with hypertension. Our results are partially consistent with those obtained for a northeastern Han Chinese population, wherein no association between *ACE2* rs2106809 and hypertension was noted^36^. Another study reported no significant relationship between *ACE2* rs2106809 and essential hypertension in a Han population in central China^37^. Liu et al. found that *ACE2* rs2074192 is associated with increased DBP, but not increased SBP^16^. However, one study has supported the dominant roles of these two *ACE2* polymorphisms in the development of hypertension^38^. We believe that this discrepancy may be due to genetic differences among the study populations. Unlike the Han population in southern China, the above-mentioned study populations were of the Uyghur group in northwest China. In a study involving multiple ethnic groups from Northwest China, the 8790A *ACE2* variant was not associated with hypertension in the Han population but was associated with an increased risk of hypertension in the Dongxiang population^39^. Genetic differences between ethnic groups lead to differential susceptibilities to disease, as do regional differences in living environments and eating habits. It is therefore necessary to establish an independent database of susceptibility genes for different ethnic groups and regions.

The present study has certain limitations. First, some environmental and lifestyle data, including diet and exercise status, were not available. Therefore, the impact of these factors on the results remains unclear. Second, genetic heterogeneity exists among different ethnic groups. Our data only included the Han Chinese population. Studies on other ethnic groups and multi-ethnic populations may help verify our results. Finally, our sample size was not sufficiently large, necessitating large multicenter studies to further determine whether *ACE2* variations can be a genetic determinant of MetS.

Our study confirmed that the rs2074192 and rs2106809 polymorphisms of *ACE2* hold promise as genetic susceptibility markers for MetS through their association with obesity. This further supports the key role of *ACE2* variants in MetS in the Chinese population, potentially enabling the early identification of individuals at a high risk of MetS. However, the findings observed between different populations need to be further validated. Large sample size, multi-ethnic design, and subgroup analysis should be evaluated in the future.

## Methods

### Study participants

In total, 339 patients with MetS and 398 non-MetS subjects, who were long-term residents of Fujian Province, China, participated in the study from 2016 to 2021. According to the Chinese guidelines for the prevention and treatment of dyslipidemia in adults^40^, the diagnostic criteria for MetS were defined as meeting three or more of the following five criteria: (1) Waist circumference (WC) > 90 cm in men or > 85 cm in women; (2) Plasma triglyceride ≥ 1.7 mmol/L; (3) Plasma high-density lipoprotein cholesterol (HDL-C) < 1.04 mmol/L, (4) Blood pressure ≥ 130/85 mmHg; (5) History of diabetes, or fasting blood glucose (FBG) ≥ 6.1 mmol/L, or 2-h postprandial blood glucose ≥ 7.8 mmol/L. Exclusion criteria included secondary hypertension, chronic heart failure, chronic glomerulonephritis, inflammatory diseases, hyperthyroidism, pulmonary heart disease, cardiac surgery, as well as the use of angiotensin-converting enzyme inhibitors, angiotensin receptor blockers, statins, and Betts. This work was approved by the Ethics Committee of the First Affiliated Hospital of Fujian Medical University (approval number: [2020] 397), and all participants signed informed consents.

### Clinical data collection and laboratory measurements

Medical history and basic information, including age, sex, history and duration of hypertension and diabetes, smoking and drinking status, as well as the history of drug use (such as the use of blood pressure and hypoglycemia medication), were obtained for all subjects.

WC, height, weight, and blood pressure were measured in all participants. WC measurements were performed using a soft ruler attached to the skin at the midpoint of the line between the anterior superior iliac crest and the 12th costal margin, without additional pressure, and with the participants' feet separated by 30 to 40 cm (shoulder width). Body mass was measured with a digital scale and the height was measured with a wall-mounted rangefinder, to the nearest 0.1 kg and 0.1 cm, respectively. Body mass index (BMI) was calculated by dividing weight (kg) by height (m) squared. These measurements were taken after fasting for 8–12 h, and participants wore light clothing and no socks or shoes. Blood pressure was measured three times at 3-min intervals while the participants remained seated with their arms supported at heart level after a 5-min rest, using a standardized automatic electronic sphygmomanometer (HBP-1300; Omron Medical, Liaoning, China). The average levels of 3 measurements of systolic blood pressure (SBP), diastolic blood pressure (DBP), and heart rate were recorded for analysis.

Blood samples were collected before breakfast after an 8–12 h overnight fast, except the 2-h postprandial plasma glucose samples, which were collected 2 h after breakfast. A completely automatic biochemical analyzer (ADVIA 2400 Chemistry System, Siemens, Japan) was used to measure total cholesterol, HDL-C, triglycerides, low-density lipoprotein cholesterol (LDL-C), very low-density lipoprotein cholesterol (VLDL-C), Apolipoprotein A1 (ApoA1), Apolipoprotein B (ApoB), fasting blood glucose, 2-h postprandial blood glucose, and glycated hemoglobin (HbA1c). These methods have been described previously^41–44^.

### Genotyping assay

*ACE2* SNPs (rs2074192, rs2106809, rs879922, and rs4646155) were selected based on human genome sequence databases and published literature. Primers and probes of *ACE2* SNPs were designed according to the sequence information (See supplementary materials, Table S2–S3) and synthesized by Shanghai General Biotechnology Co., LTD. Blood samples were collected from the forearm veins of the participants, from which genomic DNA was extracted using the TIANamp Genomic DNA kit (Tiangen Biotechnology Co., LTD., Beijing, China) according to the instructions. SNP genotyping was performed by polymerase chain reaction (PCR)-ligase detection reaction^43^, which mainly consisted of two steps: in the first step, PCR amplification conditions were 95 °C for 5 min, 94 °C for 20 s, 55 °C for 20 s, 72 °C for 40 s for 35 cycles, and, finally, at 72 °C for 10 min; in the second step, ligase detection conditions were 94 °C (20 s) and 58 °C (90 s) for 30 cycles, with a total reaction volume of 10 μl. Finally, 9 μl loading buffer was mixed with 1 μl reaction product, denatured at 95 °C for 3 min, and rapidly cooled in ice water. Fluorescent products were sequenced (3730xl DNA Analyzer; Thermo Fisher Technologies Co., LTD., USA) for measurement. PCR can rapidly and efficiently amplify specific DNA fragments. However, there are some limitations associated with the PCR. First, the fragments amplified by PCR are usually short, which limits the detection of larger DNA fragments. Second, PCR results can be affected by factors such as hybridization and primer mismatches, causing false positive or false negative results. In addition, the process of PCR amplification requires the selection of appropriate primers, which is a limitation in cases where the gene or primer is unknown.

### Statistical analysis

Statistical analysis was performed using SPSS 25.0 software (SPSS Inc.). Continuous variables were described as the mean ± standard deviation or median (interquartile spacing) and tested with Student's t-test or the Wilcoxon rank sum test. Categorical variables are presented as an absolute value (n) and a percentage (%), analyzed with chi-square tests. All statistical analyses were sex-stratified, as ACE2 was located on the X chromosome. Each SNP was tested using Hardy–Weinberg balance tests and Chi-square tests, comparing heterozygous and homozygous variant genotypes with homozygous wild-type genotypes. The extent of pairwise linkage disequilibrium between SNPs, characterized by |D '| and r^2^, was calculated by Haploview software (version 4.1; https://www.broadinstitute.org/haploview/haploview). After adjusting for confounding factors, binary logistic regression analysis was used to investigate the effects of alleles and genotypes on MetS and its components as well as to estimate the odds ratio (OR) of the risk of MetS and components with a 95% confidence interval (CI). *P* < 0.05 indicated that differences were statistically significant.

### Ethics approval and consent to participate

The study was conducted in accordance with the Declaration of Helsinki, and approved by the Ethics Committee of the First Affiliated Hospital of Fujian Medical University (approval no. [2020]397). Informed consent was obtained from all subjects involved in the study.

### Supplementary Information

Supplementary Information.

## Data Availability

The dataset used to support the findings of this study are available from the corresponding author upon request.

## Abbreviations

ACE2: Angiotensin-converting enzyme 2
MetS: Metabolic syndrome
SNPs: Single nucleotide polymorphisms
TC: Total cholesterol
TG: Total triglyceride
HDL: High-density lipoprotein cholesterol
LDL: Low-density lipoprotein cholesterol
VLDL: Very low-density lipoprotein cholesterol
HbA1c: Glycosylated hemoglobin
FPG: Fasting blood glucose
PPG: Postprandial plasma glucose
BMI: Body mass index
WC: Waist circumference
SBP: Systolic blood pressure
DBP: Diastolic blood pressure
HR: Heart rate
