# Supplementary material for: Genetic variations in ACE2 gene associated with metabolic syndrome in southern China: a case–control study
Source: Sci Rep. 2024 May 7;14:10505. doi: 10.1038/s41598-024-61254-5 (PMC11076479; doi:10.1038/s41598-024-61254-5)
Supplement: Supplementary file 1 — Supplementary Information. [file 41598_2024_61254_MOESM1_ESM.docx]

**Supplementary Materials**

**Figure S1 Linkage disequilibrium (LD) map for *ACE2* polymorphisms.**

Relative positions of *ACE2* tagSNPs were described in the upper panel. Disequilibrium coefficient |D '| expressed as a percentage for every two studied polymorphisms were shown in a triangle of cells. The r^2^ color scheme of Haploview was used (white indicated r^2^ = 0, pink indicated 0 < r^2^ <1, and red indicated r^2^ = 1).

**Table S1 Linkage disequilibrium (D' and r2) for *ACE2* tagSNPs.**

|  | rs2074192 | rs2106809 | rs4646155 | rs879922 |
| --- | --- | --- | --- | --- |
| rs2074192 |  | 0.801 | 0.498 | 0.549 |
| rs2106809 | 0.617 |  | 0.816 | 0.746 |
| rs4646155 | 0.002 | 0.023 |  | 0.829 |
| rs879922 | 0.003 | 0.023 | 0.588 |  |

The cells below the diagonally descending shaded boxes show the r^2^ values. The cells above the shaded boxes show the |D'| values of the linkage disequilibrium measurements calculated by Haploview software.

**Table S2 The primers for *ACE2* tagSNPs**

| Site |  | Primer sequence |
| --- | --- | --- |
| rs879922 | Forward primer | 5’-TCATCAGCAATCAATAGTATCAT-3’ |
|  | Reverse primer | 5’-TTAATAGCCCTTTCACATAAATG-3’ |
| rs2074192 | Forward primer | 5’-GTTCATCAACAGCTCCATT-3’ |
|  | Reverse primer | 5’-GTATAATAGAAGAGGTGTATCCAAT-3’ |
| rs2106809 | Forward primer | 5’-ATACCACAATGdCAGAGAA-3’ |
|  | Reverse primer | 5’-AAATAATAGAAGGCATCCAAACT-3’ |
| rs4646155 | Forward primer | 5’-AAGGCACAAACCACAGA-3’ |
|  | Reverse primer | 5’-CCTCCTCATTGCTCAAGA-3’ |

**Table S3 The probes for *ACE2* tagSNPs**

| ACE2 tagSNPs | Length | Probe sequence |
| --- | --- | --- |
| rs879922-TC | 25 | CCAAAAACATATGTTCTTCTCCTAC |
| rs879922-TG | 28 | TGACCAAAAACATATGTTCTTCTCCTAG |
| rs879922-TR(FAM) | 25 | TAACCCCAGTCCTTGAATTTGCTGG |
| rs2074192-TC | 30 | ACTGAGTGTGGAAATGTATAAATGCTTGGC |
| rs2074192-TT | 33 | CTGACTGAGTGTGGAAATGTATAAATGCTTGGT |
| rs2074192-TR(FAM) | 29 | ATTTATTCATTTGTGACTGCTGTGTCTGA |
| rs2106809-TA | 35 | TGACTGACTGTTTTTCCATATCTCTATCTCATGGA |
| rs2106809-TG | 38 | CTGACTGACTGACTTTTTCCATATCTCTATCTCATGGG |
| rs2106809-TR(FAM) | 33 | CTTCTCCACACTTCTACATCAGCAGACTGACTG |
| rs4646155-TA | 31 | TGACTGTCTTAACCTTGGCAAAATATACTTA |
| rs4646155-TG | 34 | CTGACTGACTCTTAACCTTGGCAAAATATACTTG |
| rs4646155-TR(HEX) | 30 | TGAATTGATTGAGACCTATCTEAAACTGAC |
